# A comparative study of three chemometrics methods combined with excitation–emission matrix fluorescence for quantification of the bioactive compounds aesculin and aesculetin in Cortex Fraxini

**DOI:** 10.3389/fchem.2022.984010

**Published:** 2022-09-07

**Authors:** Ze Ying Li, Xin Kang Li, Yuan Lin, Na Feng, Xiang-Zhi Zhang, Qing-Lin Li, Bao Qiong Li

**Affiliations:** ^1^ School of Biotechnology and Health Sciences, Wuyi University, Jiangmen, China; ^2^ Agricultural Science Research Institute of Yiyang, Yiyang, China

**Keywords:** Cortex Fraxini, excitation-emission matrix fluorescence, second-order calibration, machine learning, quantitative analysis, UPLC

## Abstract

Cortex Fraxini is an important traditional Chinese herbal medicine with various medical functions. Aesculin and aesculetin are the main effective components of Cortex Fraxini. The fluorescence signals of the two compounds have a high degree of overlap with each other, making quantitative analysis difficult with conventional analytical methods. In the present study, different chemometrics methods, including lasso regression (LAR), interval partial least squares (iPLS), and multidimensional partial least squares (N-PLS) methods, were employed and combined with excitation–emission matrix (EEM) fluorescence for the purpose of accurate quantification of aesculin and aesculetin in Cortex Fraxini samples. The most satisfactory results were obtained by using the N-PLS method based on the EEM spectra without scatterings, with correlation coefficient of calibration and prediction values higher than 0.9972 and 0.9962, respectively, root mean squared errors for calibration and prediction values lower than 0.0304 and 0.1165, respectively, and recovery values in the range of 83.32%–104.62%. The obtained credible models indicated that the N-PLS method combined with EEM spectra has the advantages of being green, low cost, and accurate and it is a good strategy for the determination of active compounds in complex samples. To further confirm the accuracy of the obtained results, the same samples were analyzed by the recognized ultra-performance liquid chromatography method.

## 1 Introduction

Chinese herbal medicine is a natural resource with abundant sources. It is widely used in the treatment of diseases in China and neighboring countries. Compared with western medicine, Chinese herbal medicine is popular with people because of its low toxicity and few side effects. Cortex Fraxini (Chinese name Qin-pi) is an important traditional Chinese herbal medicine that has been used to treat gout, arthritis, hyperuricemia, and other diseases (Wang et al., 2016a) for over 2000 years. Coumarin compounds are the main chemical active ingredients in Cortex Fraxini medicinal materials, which have obvious effect in the treatment of primary hyperuricemia (Zhou et al., 2018).

Nowadays, numerous methods exist for the quantitative analysis of target ingredients of Chinese herbal medicines or their formulas, including high-performance liquid chromatography (HPLC), ultra-performance liquid chromatography (UPLC), liquid chromatography–mass spectrometry (LC-MS), gas chromatography–mass spectrometry (GC-MS), ultra-high-performance supercritical fluid chromatography (UHPSFC), Fourier-transform infrared (FTIR) spectroscopy, excitation–emission matrix (EEM) fluorescence spectroscopy, terahertz (THz) spectroscopy, and proton nuclear magnetic resonance (^1^H NMR) spectroscopy. Among these methods, EEM spectroscopy has the advantages of rapidity, simplicity, low cost, high sensitivity, and non-destructiveness. At present, a large number of reports on the analysis of ingredients in foods and medicines by fluorescence spectroscopy combined with chemometrics methods have been reported, and a few examples include those of Bai et al. (2018), Liu et al. (2019), Wang et al. (2017), and Li et al. (2021). These successful examples demonstrated that fluorescence spectroscopy combined with chemometrics methods can be regarded as an effective way to quantitatively analyze target components in complex systems.

Aesculin and aesculetin are the main effective components of Cortex Fraxini. At present, many analytical methods have been applied to analyze one or both components of aesculin and aesculetin, including HPLC (Wang et al., 2019), LC-MS (Li et al., 2013), capillary electrophoresis (CE) (Li et al., 2005), GC-MS (Wang et al., 2016b), and electrochemical analysis (Sheng et al., 2020). In our research, we intend to quantitatively analyze these two components based on EEM spectra because of its advantages of simplicity, low cost, and high sensitivity. Owing to the complexity of the Cortex Fraxini samples, unknown interferences may be present in the measured data, and the fluorescence signals of aesculin and aesculetin have a high degree of overlap with each other, making simultaneous quantitative analysis difficult by using conventional fluorescence methods. To realize the accurate quantitative analysis purposes, appropriate algorithms should be explored and employed.

The aim of this work was to explore different chemometrics strategies, such as lasso regression (LAR), interval partial least squares (iPLS), and multidimensional partial least squares (N-PLS) methods, in combination with EEM spectra for the quantitative determination of aesculin and aesculetin in Cortex Fraxini samples. Among these three methods, LAR is an important machine learning technique that can be applied for analyzing data suffering from multicollinearity (Gao et al., 2022), iPLS is a feature interval extraction method on the basis of PLS that aims to minimize multicollinearity problems to improve the accuracy of the PLS model (Abreu et al., 2015), and N-PLS is a second-order multivariate calibration algorithm that can be used for three-way data modeling even in the presence of multicollinearity problems (Lopez-Fornieles et al., 2022). To the best of our knowledge, there exist no published reports of the analysis of the aesculin and aesculetin in Cortex Fraxini samples by using the above-mentioned methods. The obtained results of each method were fully validated by statistical parameters, and the potentials of the methods were evaluated and compared. The comparison results showed that the N-PLS method gave the most satisfactory quantitative analysis results. Furthermore, we compared the most satisfactory results achieved from the N-PLS method with those obtained by the UPLC method, which further proved the reliability and accuracy of the explored method.

## 2 Theory

### 2.1 Chemometrics methods

In the following subsections, a brief introduction is given to the three employed methods, LAR, iPLS, and N-PLS.

#### 2.1.1 Lasso regression (LAR)

The LAR method is an important machine learning technique for analyzing data suffering from multilinearity (Yang and Wen, 2018). The basic idea of LAR is to minimize the sum of squared residuals under the constraint that the sum of the absolute values of the regression coefficients is less than a constant, so that some regression coefficients strictly equal to zero can be generated and an interpretable model can be obtained (Tkachenko et al., 2021) when the regression coefficients are zero and the corresponding variables are not selected, thus eliminating irrelevant information variables and improving the predictive accuracy of the model and its interpretability.

#### 2.1.2 Interval partial least squares (iPLS) algorithm

The iPLS algorithm can be used to extract the feature bands and remove a large amount of useless information and noisy variables. It divides the entire spectral region into several subintervals of equal width. For each interval, a local PLS is established. If a subinterval contains more information and less noise, the performance of the corresponding model will be better (Wu et al., 2015). Therefore, in each subinterval, the root mean squared errors for cross-validation (*RMSECV*) are calculated, and the subinterval with the minimum *RMSECV* value is taken as the characteristic band and selected for further use. For cross-validation, an individual sample is taken from the calibration set and a model is established by the remaining samples. Then, the model is applied to predict the removed sample. The calculation formula of *RMSECV* is as follows (Zhang et al., 2021):

$$RMSECV=\sqrt{\frac{\sum_{i=1}^{m}{\left(y_{i}-{\tilde{y}}_{i}\right)}^{2}}{m}},$$ (1)

where *m* is the sample numbers of the calibration set, $y_{i}$ is the reference value of sample *i*, and ${\tilde{y}}_{i}$ is the calculated value of the removed sample *i*.

#### 2.1.3 Multidimensional partial least squares (N-PLS)

N-PLS (Bro, 1996) is an extension of partial least squares (PLS) regression to higher-order arrays; it has been used in many quantitative analysis studies (Bro, 1996; Luo et al., 2016). During the construction of the N-PLS models, it is important to choose the proper number of latent variables (LVs). Some necessary and useful information may be missed if the number of LVs is too small, whereas some useless information or even interferences will be involved in the model if the number of LVs is more than required (Yang et al., 2018). Therefore, the proper number of LVs should be determined carefully. In this study, the proper number of LVs was chosen on the basis of the minimum predicted residual error sum of squares (*PRESS*) for each model (Thomas, 2003). The calculation formula of *PRESS* is as follows (Khajehsharifi and Eskandari, 2011):

$$PRESS=\sum_{i=1}^{m}{\left(y_{i}-{\hat{y}}_{i}\right)}^{2},$$ (2)

where *m* is the sample numbers of the calibration set and $y_{i}$ and ${\hat{y}}_{i}$ are the reference and calculated values, respectively, of sample *i* in the calibration samples.

### 2.2 Validation of model performance

The performance of the established models should be validated by statistical parameters. For the above purpose, the following parameters have been employed: the correlation coefficient for calibration (*Rc*) and prediction (*Rp*) and the root mean squared errors for calibration (*RMSEC*) and prediction (*RMSEP*). The correlation coefficient is a measure of the linearity between the calculated and actual values of the model, and the root mean squared error is a commonly used index to measure the error in quantitative models. A successful calibration model should have higher values of *Rc* and *Rp* and lower values of *RMSEC* and *RMSEP*.

## 3 Experimental

### 3.1 Reagents and solutions

**FIGURE 1:**
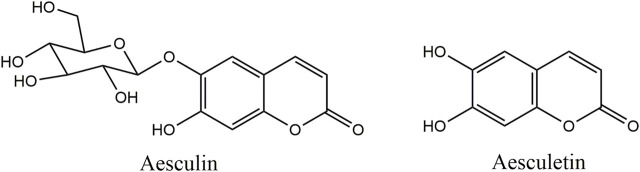
Molecular structures of aesculin and aesculetin.

Aesculin and aesculetin were purchased from Chengdu MUST BIO-TECHNOLOGY CO., LTD. (purity higher than 98%). The molecular structures of the two compounds are illustrated in Figure 1. Ultra-pure water was purchased from Watsons. Methanol was of HPLC grade and other chemicals were of analytical grade.

Six Cortex Fraxini samples were purchased from local drug stores (Jiangmen, Guangdong, China) and then pulverized with a traditional Chinese medicine grinder.

### 3.2 Apparatus

Fluorescence data of all samples were measured by a fully automatic and integrated FluoroMax-4 instrument with a 150 w ozone-free xenon light source, excitation monochromator, reference detector, sample cell, emission monochromator, and signal detector. The spectral data were obtained by scanning the samples at excitation wavelengths ranging from 200 nm to 600 nm and emission wavelengths ranging from 200 nm to 600 nm with steps of 10 nm.

The multifunctional ultrasonic cleaner (GS-060A) was purchased from Shenzhen Keneng Cleaning Equipment Co., Ltd.

The UPLC chromatograms were carried out on Waters UPLC H-class instrument coupled with a PAD e λ detector, a sample manager, a quaternary solvent manager, and a BEH C18 column (2.1 × 100 mm, 1.7 μm). The gradient elution program used 0.1% v/v formic acid in water (A) and acetonitrile (B) as the eluents, and the program was as follows: 0–1.00 min, 13%–15% B; 1.00–5.00 min, 15%–20% B; 6.00–9.00 min 100% B; 9.00–10.00 min 100%–13.00% B; 10.00–12.00 min 13% B. The column temperature was at room temperature, the scan wavelength ranged from 210 to 400 nm, the flow rate was set at 0.5 ml/min, and the injection volume was 10 μl (Preeti Chandra et al., 2016).

The implementation of the N-PLS and iPLS algorithms was on the eclipse platform, and the version number was 4.19.0; the jdk version was 1.7.0_13-b20, the tomcat version was 7.0, and the computer language was java. The implementation of the LAR algorithm was on the pycharm platform, and the computer language was python.

### 3.3 Sample preparation

Stock solutions of aesculin (745 μg/ml) and aesculetin (1,060 μg/ml) were prepared by dissolving the standards in methanol, and they were stored at 4°C in the freezer. Working solutions were prepared by appropriate dilution of the stock solutions with methanol. Britton–Robinson buffer solution (BR buffer) (0.2 mol/L, pH = 9) was used to stabilize the fluorescence intensity of aesculin and aesculetin.

A series of 21 mixed standard solution samples was prepared. The concentrations of aesculin and aesculetin were in the range of 0.002–1.020 μg/ml and 0.009–4.000 μg/ml, respectively, and the detailed concentrations of the aesculin and aesculetin are shown in Supplementary Tables S1, S2, respectively (in the Supporting information).

The purchased Cortex Fraxini samples were crushed into powder by a pulverizer for better extraction of aesculin and aesculetin. We referred to some Chinese literature and found that the active substances in Cortex Fraxini can be better extracted by soaking the sample in methanol for 24 h and then extracting by ultrasonication for 30 min. Thus, in this study, the powdered Cortex Fraxini samples were soaked in methanol for 24 h at room temperature and then treated with ultrasonication for 30 min. They were then filtered and transferred to a 50.0 ml volumetric flask, left at room temperature, and appropriately diluted with methanol during use.

For the recovery test, the six actual samples were all spiked with a certain amount of aesculin and aesculetin, respectively. The recovery values were calculated as $\left(Con_{1}-Con_{0}\right)/Con_{\text{s}}$ , where *Con*_1_ was the calculated concentration of spiked samples, *Con*_0_ was the calculated concentration of unspiked samples, and *Con*_s_ was the concentration of the target compounds added to real samples.

For all of the above samples, 2 ml of BR buffer was added and then the samples were diluted to the mark in 10.0 ml volumetric flasks with ultra-pure water. Each mixed standard sample was measured once, and each spiked and actual sample was measured three times to obtain the average value.

## 4 Results and discussion

### 4.1 Spectral characters

**FIGURE 2:**
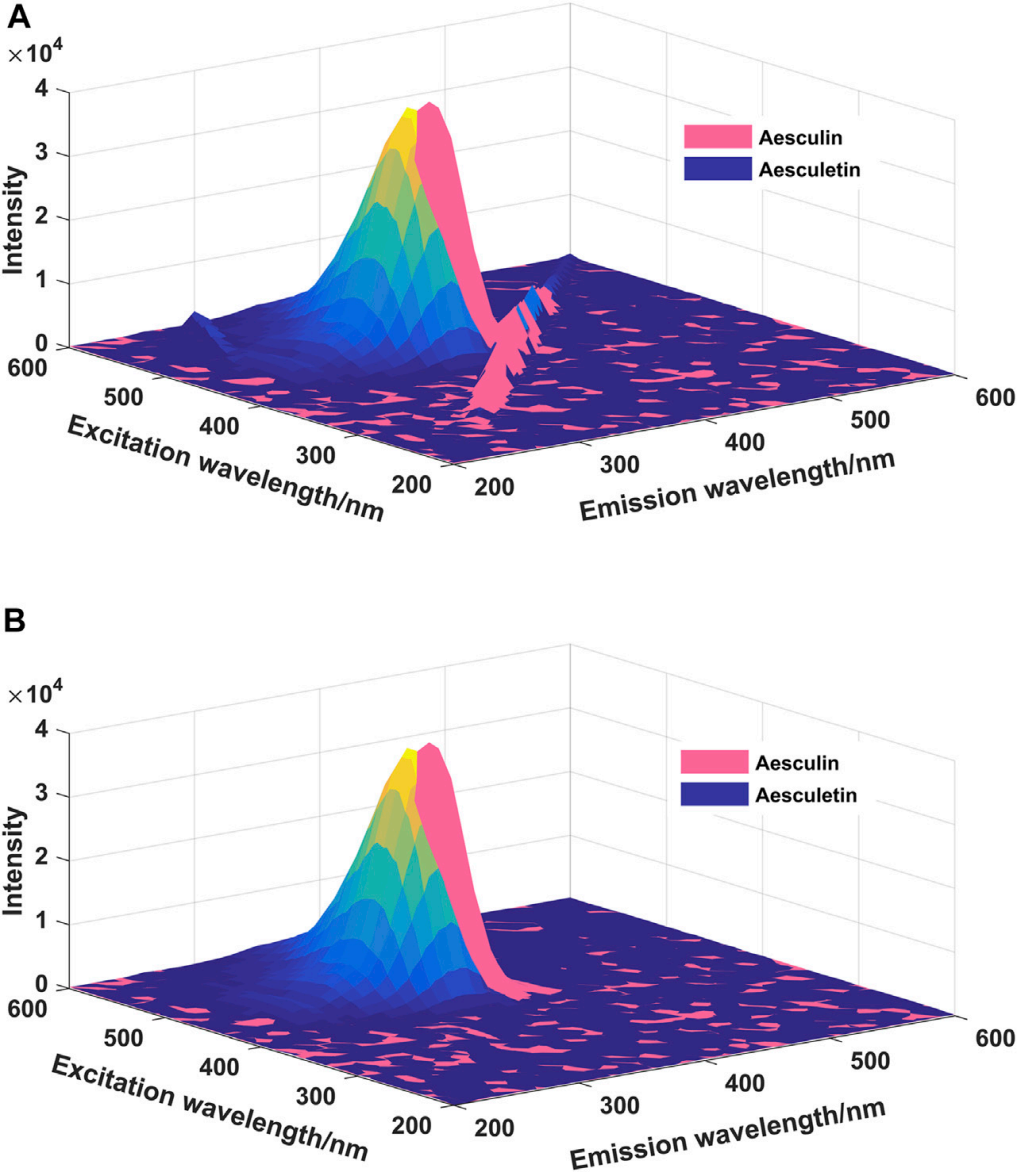
The EEM spectra of aesculin (0.16 μg/ml) and aesculetin (1 μg/ml). **(A)**: Scatterings are not eliminated; **(B)**: Scatterings are eliminated.

In EEM spectra, Raman and Rayleigh scatterings are unrelated to the chemical sample composition (Andersen and Bro, 2003), and the existence of Raman and Rayleigh scatterings may have some influence on the final analytical results. The EEM spectra of aesculin and aesculetin are presented in Figure 2; Figure 2A is with Raman and Rayleigh scatterings, and Figure 2B is without scatterings. Moreover, one can see that there is a high degree of overlap between the two signals. Therefore, it is difficult to determine the concentration of the two components by the ordinary fluorescence method. In the present study, the scatterings are first removed and then the missing data is filled by interpolation by using new data consistent with the rest of the EEM spectrum (Bahram et al., 2006). As can be seen from Figure 2B, the scatterings have been removed successfully. In order to obtain satisfactory analytical results, in the present study, three well-known chemometrics methods, namely LAR, iPLS, and N-PLS, were employed, and the results obtained are compared and discussed herein.

The EEM spectra matrices without scatterings were arranged in a three-way array with the dimensions of 41 (emission wavelength points) × 41 (excitation wavelength points) × sample numbers. To explore the main characteristics of the employed methods, the mixed standard samples were divided into calibration and prediction sets; three quarters of the samples were selected as the calibration set and used to establish the calibration model, and the remaining ones were selected as the prediction set and applied to validate the performance of the established model. For the calibration set, the matrix size was 41 × 41 × 16, and for the prediction set the matrix size was 41 × 41 × 5.

### 4.2 Results and discussion

A suitable method was the vital factor for model establishment. Three algorithms, namely LAR, iPLS, and N-PLS, were applied individually to build calibration models. The performances of the established models were compared with each other by evaluation of *Rc*, *Rp*, *RMSEC*, and *RMSEP*.

#### 4.2.1 LAR models

The first method explored was the LAR method. The existence of multicollinearity may cause the model to shift greatly and cannot simulate the overall view of data. LAR is an important machine learning technique for analyzing data that suffer from multilinearity. Before the LAR model establishment, the contour values (Li et al., 2015) of the EEM spectra of all samples were extracted for dimensionality reduction purposes. The process of extracting the contour values is equivalent to the process of extracting the emission wavelength values by fixing the excitation wavelength. A plot of the contour values of the EEM spectrum of a sample is presented in Supplementary Figure S2. After extraction of the contour values, the data matrix of the calibration set had a size of 16 (sample number) × 41 (emission wavelength points). For the prediction set, the matrix had a size of 5 (sample number) × 41 (emission wavelength points). By application of this method, the irrelevant information variables was eliminated and a regression model was obtained for each target compound. In this case, the LAR model for each target compound was described as follows:

The LAR model for aesculin:

$$\begin{array}{l} C_{\mathrm{Aesculim}}=-2.89\times 10^{-2}+8.10\times 10^{-2}\times V_{200}+3.00\times 10^{-6}\times V_{210}-8.00\times 10^{-6}\times V_{220}\\ \;\;\;+3.90\times 10^{-5}\times V_{250}+5.20\times 10^{-5}\times V_{280}+2.00\times 10^{-6}\times V_{410}+1.10\times 10^{-5}\times V_{450}\\ \;\;\;-3.29\times 10^{-4}\times V_{470}+1.34\times 10^{-4}\times V_{480}+6.00\times 10^{-6}\times V_{520}+8.70\times 10^{-5}\times V_{530}\\ \;\;\;-3.57\times 10^{-4}\times V_{560}+3.80\times 10^{-4}\times V_{570} \end{array}.$$ (3)

The LAR model for aesculetin:

$$\begin{array}{l} C_{\mathrm{Aesculetin}}=-4.02+1.50\times 10^{-5}\times V_{260}+4.60\times 10^{-5}\times V_{270}+4.19\times 10^{-4}\times V_{430}\\ \;\;\;+9.29\times 10^{-4}\times V_{490} \end{array}.$$ (4)

In the above models, $C_{Aesculin}$ and $C_{Aesculetin}$ were the concentrations of aesculin and aesculetin, respectively, $V_{200}$ was the contour value at an emission wavelength of 200 nm, and the remaining parameters followed the same pattern in their definitions.

**FIGURE 3:**
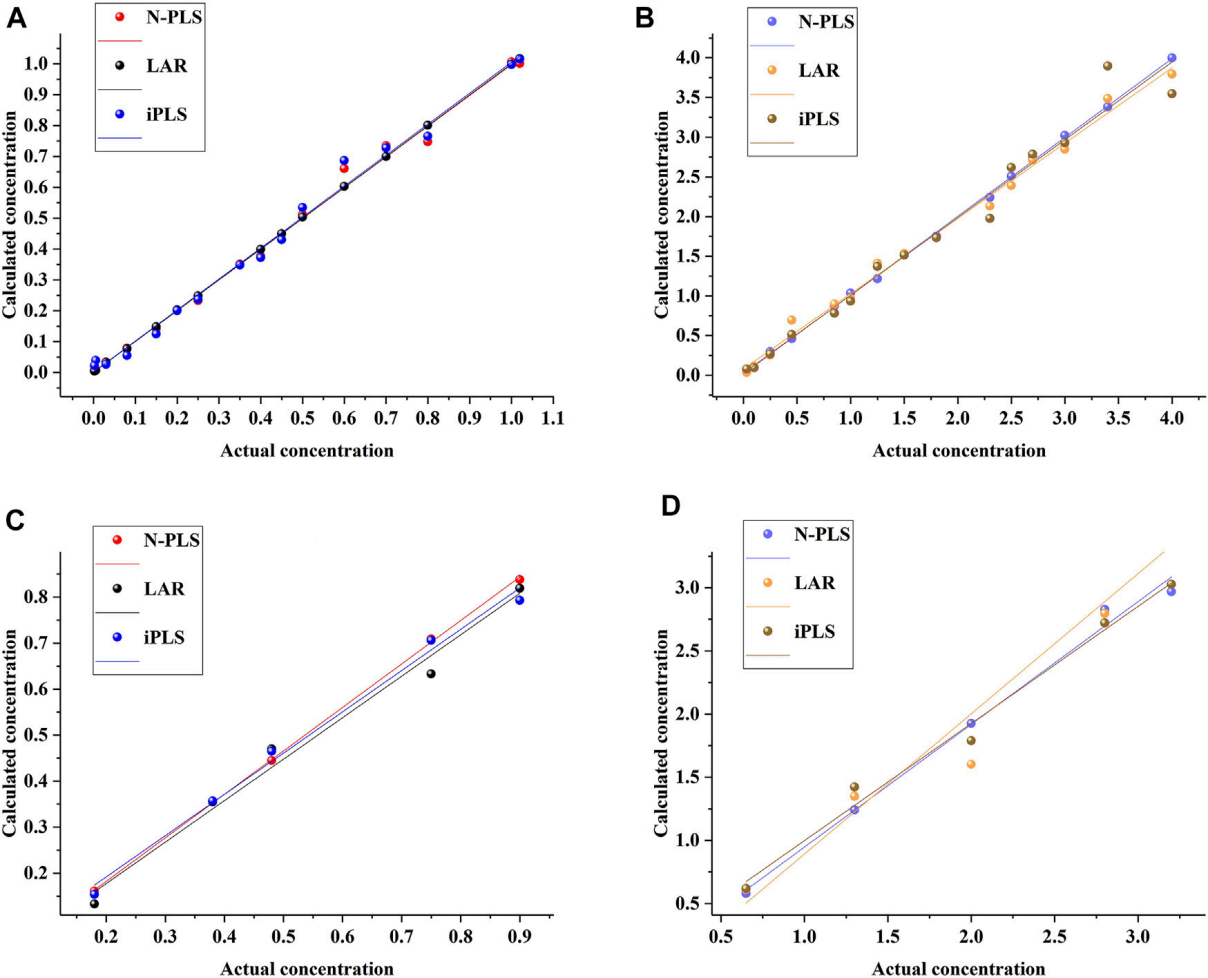
The relationship between the actual concentrations and calculated concentrations by using different methods for aesculin **(A)** and aesculetin **(B)** in the calibration set and aesculin **(C)** and aesculetin **(D)** in the prediction set.

To assess the prediction ability of the established models, they were estimated by prediction sets; the statistical results are shown in Table 1. As can be seen, the values of *Rc* were 1.0000 and 0.9967 and of *RMSEC* were 0.0023 and 0.1174 for aesculin and aesculetin, respectively, demonstrating that the established models have good linearity and can be applied to the prediction set. For prediction purposes, *Rp* values of 0.9921 and 0.9767 (Figure 3) and *RMSEP* values of 0.0680 and 0.2507 were obtained for aesculin and aesculetin, respectively.

**TABLE 1:** Statistical parameters related to the calibration and prediction models of aesculin and aesceletin established by different methods.

Analytical method	Analyte	Calibration set		Prediction set	
		*Rc*	*RMSEC*	*Rp*	*RMSEP*
LAR	Aesculin	1.0000	0.0023	0.9921	0.0680
iPLS		0.9958	0.0307	0.9962	0.0544
N-PLS		0.9972	0.0243	0.9998	0.0392
LAR	Aesculetin	0.9967	0.1174	0.9767	0.2507
iPLS		0.9875	0.1969	0.9940	0.1386
N-PLS		0.9997	0.0304	0.9962	0.1165

LAR: lasso regression.

iPLS: interval partial least squares.

N-PLS: multidimensional partial least squares.

In addition, as can be seen from Table 2, the recovery values were in the range of 80.04%–103.36%, and 76.65%–106.03% for aesculin and aesculetin, respectively. One can conclude that not all recovery results were satisfactory, such as the recovery of aesculetin (76.65%) obtained from Cortex Fraxini 4. We suspect the possible reason is that the unknown interference and useless information in the spectra has an impact on the final analysis results. Therefore, as described in the following section, a method that can be used to extract characteristic variables to improve the accuracy of the analytical results was explored.

**TABLE 2:** Recovery values obtained by different methods.

Samples	Analyte	Spike (μg/ml)	Recovery (%)			
			LAR	iPLS	N-PLS	UPLC
Cortex Fraxini 1	Aesculin	0.48	103.36	96.68	90.53	102.35
Cortex Fraxini 2		0.50	96.80	99.52	99.56	92.14
Cortex Fraxini 3		0.48	102.13	100.17	99.95	81.96
Cortex Fraxini 4		0.48	80.04	82.36	88.16	96.96
Cortex Fraxini 5		0.25	99.57	105.45	103.99	86.43
Cortex Fraxini 6		0.40	89.12	90.60	87.22	89.42
Cortex Fraxini 1	Aesculetin	2.00	89.35	87.9	90.25	86.59
Cortex Fraxini 2		2.30	93.74	84.57	89.84	99.55
Cortex Fraxini 3		2.00	103.47	92.13	83.32	95.31
Cortex Fraxini 4		2.00	76.65	74.88	104.62	89.14
Cortex Fraxini 5		1.00	106.03	99.87	92.55	84.88
Cortex Fraxini 6		1.50	95.32	91.53	101.53	89.62

#### 4.2.2 iPLS models

The iPLS method can be used to extract the characteristic intervals from the given spectral data to obtain more accurate analytical results. In the present study, the iPLS method was also performed on the contour values, and 8 intervals were determined for further analysis. Then, one optimum interval and corresponding PLS component was determined according to the minimum *RMSECV* value. In the present study, the interval in the emission wavelength range of 310–350 nm is determined for both aesculin and aesculetin, and the optimization numbers of the PLS component are 2 and 1, respectively. The corresponding statistical parameters of the established iPLS models are summarized in Table 1.

As can be seen from Table 1, the iPLS models have higher *Rp* values and lower *RMSEP* values than that of LAR models, demonstrating that the iPLS method has good feature extraction ability and can be applied to improve the accuracy of the established models.

After model evaluation, the recovery values were also calculated; as can be seen from Table 2, the recovery values were in the range of 82.36%–105.45%, and 74.88%–99.87% for aesculin and aesculetin, respectively. The same phenomenon occurred as with the LAR method in that the recovery of aesculetin (74.88%) in Cortex Fraxini 4 was not satisfactory. From this perspective, we suspect that one possible reason was that some important information from the contour values might be lost, so a high-order chemometrics method was considered to deal with the EEM spectral data.

#### 4.2.3 N-PLS models

The calibration set with the size of 41 × 41 × 16 was applied to establish the calibration model, and the performance of the established models was evaluated by using the prediction set. In the process of establishing the N-PLS models, the optimal number of LVs should first be determined. In the present study, the optimum number of LVs for the N-PLS models was indicated by the minimum *PRESS* value versus the number of LVs (Supplementary Figure S1). For aesculin (Supplementary Figure S1A), the minimum *PRESS* value can be determined with 2 LVs, and for aesculetin (Supplementary Figure S1B), the local minimum *PRESS* value can be determined with 5 LVs. The statistical parameters of the N-PLS models are summarized in Table 1.

As can be seen from Table 1, the results from the calibration set show that the N-PLS method yielded satisfactory *Rc* values of 0.9972 and 0.9997 and *RMSEC* values of 0.0243 and 0.0304 for aesculin and aesculetin, respectively, demonstrating that the established models have the ability to accurately calculate the prediction set. The established models were then applied to the prediction set, and values were obtained for *Rp* of 0.9998 and 0.9962, and *RMSEP* of 0.0392 and 0.1165 for aesculin and aesculetin, respectively, which proved that good predictions were obtained.

Next, the spike recovery experiment was performed. As can be seen from Table 2, the recovery values were in the range of 87.22%–103.99% for aesculin, and 83.32%–104.62% for aesculetin. Compared with the results of the previous LAR and iPLS methods, the linearity, predictive ability, and accuracy of the N-PLS models were improved. However, owing to the same phenomenon that occurred with the LAR and iPLS methods, the recovery values of aesculetin in Cortex Fraxini 4 were nearly the same and not satisfactory, so we decided that it was necessary to further verify which method gives the most reliable recovery values.

### 4.3 UPLC method for validation

A UPLC method was developed to further confirm the accuracy and reliability of the presented strategies. The two analytes and the backgrounds can be separated properly and completely under the chromatographic conditions mentioned above, and thus they can be quantified accurately based on peak areas. The chromatograms of aesculin, aesculetin, and a Cortex Fraxini sample are shown in Supplementary Figure S3. For a comprehensive comparison, the concentrations of aesculin and aesculetin used for UPLC and fluorescence were the same. In the measurement process, the samples with lower concentrations were not detected (Supplementary Tables S1, S2); thus, the calibration equations of aesculin and aesculetin were established from samples 1 to 14 in the calibration set. The statistical parameters of the calibration equations are summarized in Table 3; the low *RMSEC* and *RMSEP* values and high *R*_*c*_ and *R*_*p*_ values illustrate the accuracy of the UPLC method. The recoveries of aesculin and aesculetin by UPLC are shown in Table 2. First, we can see that the recovery rates of aesculin (81.96%–102.35%) and aesculetin (84.88%–99.55%) were satisfactory. One can then conclude that the accuracy of the UPLC and N-PLS methods is comparable. The separation of complex samples with UPLC requires some effort to find suitable conditions. A comparison of the comprehensive results of the N-PLS and UPLC methods shows that EEM fluorescence coupled with the chemometrics method can be regarded as a reliable and accurate approach to determine the two components in Cortex Fraxini samples simultaneously.

**TABLE 3:** Analytical results related to the calibration and prediction results for aesculin and aesculetin based on the UPLC method.

Analyte	Calibration set			Prediction set	
	*R*_*c*_	*RMSEC*	Equation	*R*_*p*_	*RMSEP*
Aesculin	0.9923	0.0274	*y* = 36228*x*-797.31	0.9957	0.0816
Aesculetin	0.9858	0.1414	*y* = 53779*x*-6536.4	0.9965	0.0222

*y*: peak area.

*x*: concentration (μg/ml).

### 4.4 Further discussion

The above comparisons show that the analytical results of the N-PLS method on the EEM spectra are more accurate and reliable than the LAR and iPLS methods on the contour values. The reason may be that the higher-order N-PLS algorithm is less disturbed by the noise of the analysis system, so the quantitative analysis results obtained are more accurate (Pierce et al., 2011). Although the LAR and iPLS methods have not yielded satisfactory results, these strategies still have application values in proper analytical systems.

## 5 Conclusion

The present study reported a comparative study of three different algorithm (LAR, iPLS, and N-PLS) methods in combination with EEM spectroscopy for the determination of aesculin and aesculetin in traditional Chinese medicine. Satisfactory results were obtained with the N-PLS method on the basis of the EEM spectra despite the strong overlaps of the two analytes. The results presented herein show that the methodology proposed has the advantages of being simple, sensitive, and low cost. Consequently, EEM with the aid of proper chemometrics methods is a useful tool in processing the serious problem of overlapping and can be extended to other complex systems in the fields of food and the environment, such as body fluids.

## Data Availability

The original contributions presented in the study are included in the article/Supplementary Materials; further inquiries can be directed to the corresponding author.
