# Supplementary material for: A comparative study of three chemometrics methods combined with excitation–emission matrix fluorescence for quantification of the bioactive compounds aesculin and aesculetin in Cortex Fraxini
Source: Front Chem. 2022 Sep 7;10:984010. doi: 10.3389/fchem.2022.984010 (PMC9490370; doi:10.3389/fchem.2022.984010)
Supplement: Supplementary file 1 [file DataSheet1.docx]

**A comparative study of three chemometrics methods combined with excitation-emission matrix fluorescence for quantification of the bioactive compounds Aesculin and Aesculetin in Cortex Fraxini**

**Ze Ying Li^1^, Xin Kang Li^1^, Yuan Lin^1^, Xiang-Zhi Zhang^1^, Qing-lin Li^2^, Bao Qiong Li^1^***

^1^Wuyi University, School of Biotechnology and Health Sciences, Jiangmen, PR China

^2^Agricultural Science Research Institute of Yiyang, Yiyang, PR China

*** Corresponding authors:**

Bao Qiong Li

libq201406@163.com

Supplementary Information

**Figure captions:**

**Figure S1**The estimating of the proper number of LVs for N-PLS model: A for aesculin and B for aesculetin.

**Figure S2** The plot of contour value of the EEM spectrum of a sample

**Figure S3** The UPLC chromatograms.

**Table captions:**

**Table S1** Concentrations of the aesculin in the calibration set and prediction set

**Table S2** Concentrations of the aesculetin in the calibration set and prediction set

**Fig. S1** The estimating of the proper number of LVs for N-PLS model: A for aesculin and B for aesculetin

**
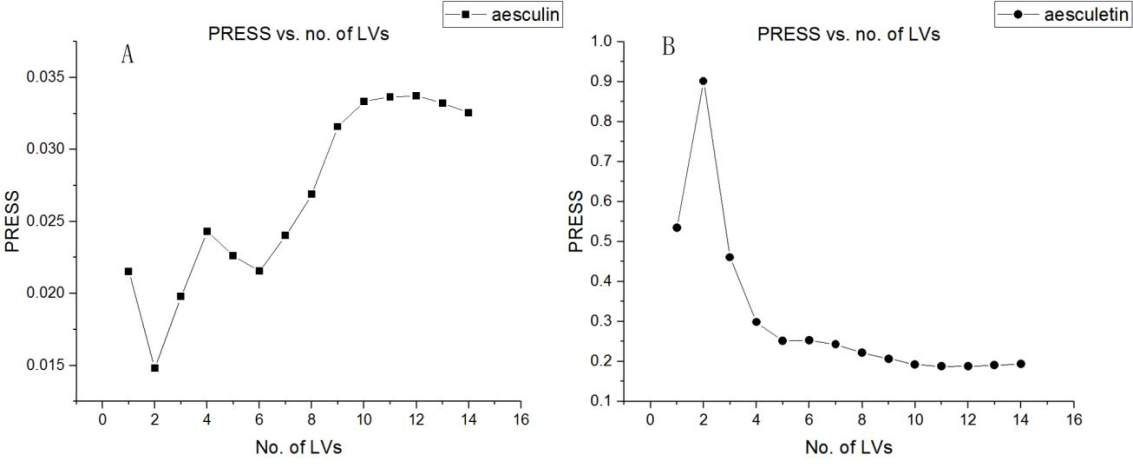
**

**Fig. S2** The plot of contour value of the EEM spectrum of a sample

**
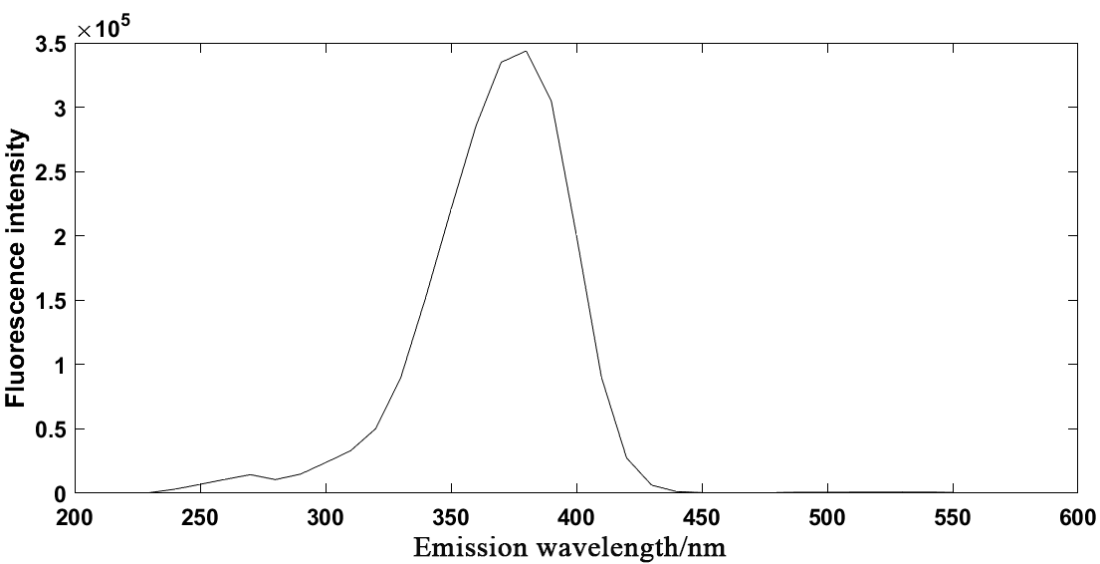
**

**Fig. S3** The UPLC chromatograms.

**
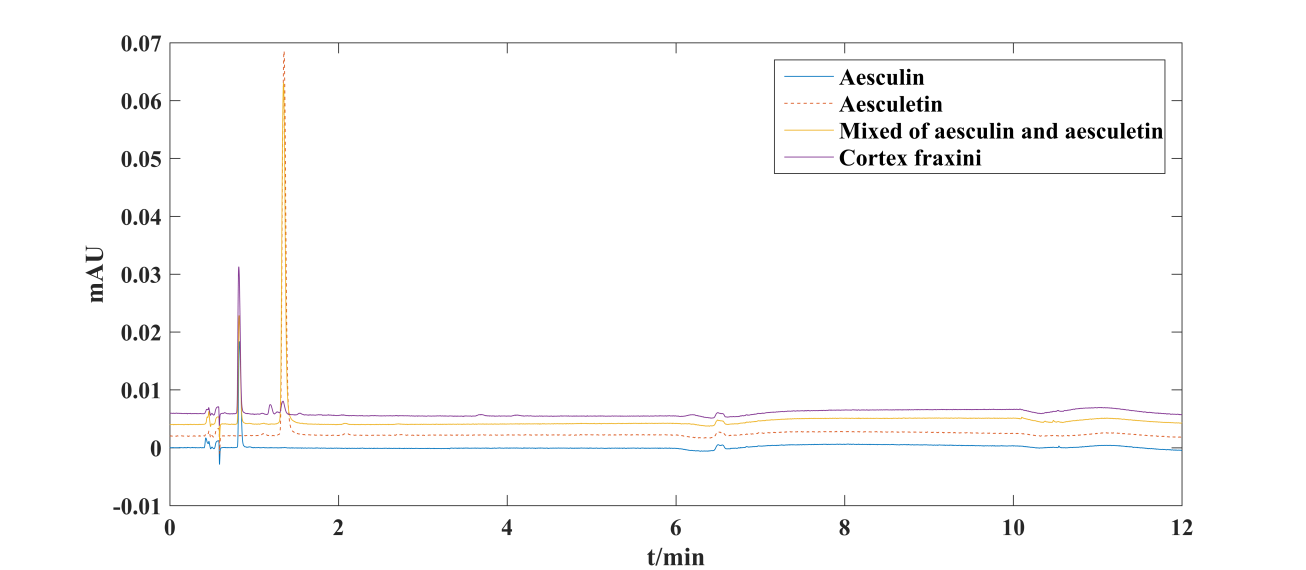
**

**Table S1** Concentrations of the aesculin in the calibration set and prediction set

| Dataset | Sample | Exp. Con. |  | Cal. Con. | | | |
| --- | --- | --- | --- | --- | --- | --- | --- |
|  |  | Aesculin |  | Aesculin | | | |
|  |  |  |  | LAR | iPLS | N-PLS | UPLC |
| Calibration set | 1 | 1.020 |  | 1.017 | 1.016 | 1.001 | 1.033 |
|  | 2 | 1.000 |  | 0.999 | 0.998 | 1.007 | 0.979 |
|  | 3 | 0.800 |  | 0.801 | 0.766 | 0.748 | 0.853 |
|  | 4 | 0.700 |  | 0.700 | 0.728 | 0.735 | 0.729 |
|  | 5 | 0.600 |  | 0.603 | 0.687 | 0.661 | 0.541 |
|  | 6 | 0.500 |  | 0.504 | 0.535 | 0.512 | 0.501 |
|  | 7 | 0.450 |  | 0.450 | 0.430 | 0.430 | 0.430 |
|  | 8 | 0.400 |  | 0.399 | 0.372 | 0.377 | 0.370 |
|  | 9 | 0.350 |  | 0.348 | 0.348 | 0.351 | 0.358 |
|  | 10 | 0.250 |  | 0.248 | 0.237 | 0.233 | 0.227 |
|  | 11 | 0.200 |  | 0.203 | 0.201 | 0.200 | 0.199 |
|  | 12 | 0.150 |  | 0.148 | 0.125 | 0.141 | 0.160 |
|  | 13 | 0.080 |  | 0.076 | 0.055 | 0.079 | 0.100 |
|  | 14 | 0.030 |  | 0.032 | 0.026 | 0.034 | 0.050 |
|  | 15 | 0.005 |  | 0.006 | 0.039 | 0.010 | --- |
|  | 16 | 0.002 |  | 0.004 | 0.022 | 0.007 | --- |
|  |  |  |  |  |  |  |  |
| Prediction set | 1 | 0.900 |  | 0.819 | 0.793 | 0.838 | 0.895 |
|  | 2 | 0.750 |  | 0.633 | 0.706 | 0.709 | 0.720 |
|  | 3 | 0.480 |  | 0.470 | 0.465 | 0.445 | 0.441 |
|  | 4 | 0.380 |  | 0.355 | 0.357 | 0.355 | 0.382 |
|  | 5 | 0.180 |  | 0.133 | 0.154 | 0.161 | 0.184 |

---: Not detected; N-PLS: Multidimensional partial least squares;

LAR: Lasso Regression; iPLS: interval partial least squares

**Table S2** Concentrations of the aesculetin in the calibration set and prediction set

| Dataset | Sample | Exp. Con. |  | Cal. Con. | | | |
| --- | --- | --- | --- | --- | --- | --- | --- |
|  |  | Aesculetin |  | Aesculetin | | | |
|  |  |  |  | LAR | iPLS | N-PLS | UPLC |
| Calibration set | 1 | 4.000 |  | 3.796 | 3.547 | 4.000 | 4.125 |
|  | 2 | 3.400 |  | 3.487 | 3.895 | 3.382 | 3.102 |
|  | 3 | 3.000 |  | 2.849 | 2.929 | 3.023 | 3.277 |
|  | 4 | 2.700 |  | 2.715 | 2.785 | 2.744 | 2.823 |
|  | 5 | 2.500 |  | 2.391 | 2.618 | 2.509 | 2.326 |
|  | 6 | 2.300 |  | 2.132 | 1.977 | 2.243 | 2.254 |
|  | 7 | 1.800 |  | 1.733 | 1.735 | 1.752 | 1.825 |
|  | 8 | 1.500 |  | 1.533 | 1.513 | 1.516 | 1.562 |
|  | 9 | 1.250 |  | 1.411 | 1.370 | 1.215 | 1.057 |
|  | 10 | 1.000 |  | 0.971 | 0.935 | 1.035 | 0.982 |
|  | 11 | 0.850 |  | 0.897 | 0.782 | 0.874 | 0.842 |
|  | 12 | 0.450 |  | 0.695 | 0.514 | 0.462 | 0.450 |
|  | 13 | 0.250 |  | 0.255 | 0.267 | 0.296 | 0.287 |
|  | 14 | 0.100 |  | 0.115 | 0.094 | 0.106 | 0.188 |
|  | 15 | 0.030 |  | 0.036 | 0.079 | 0.053 | --- |
|  | 16 | 0.009 |  | 0.123 | 0.080 | 0.028 | --- |
|  |  |  |  |  |  |  |  |
| Prediction set | 1 | 3.200 |  | 3.591 | 3.027 | 2.969 | 3.189 |
|  | 2 | 2.800 |  | 2.797 | 2.721 | 2.828 | 2.939 |
|  | 3 | 2.000 |  | 1.603 | 1.790 | 1.927 | 1.950 |
|  | 4 | 1.300 |  | 1.349 | 1.423 | 1.241 | 1.252 |
|  | 5 | 0.65 |  | 0.613 | 0.619 | 0.581 | 0.555 |

---: Not detected; N-PLS: Multidimensional partial least squares;

LAR: Lasso Regression; iPLS: interval partial least squares
